# Ulinastatin Suppresses Burn-Induced Lipid Peroxidation and Reduces Fluid Requirements in a Swine Model

**DOI:** 10.1155/2013/904370

**Published:** 2013-04-24

**Authors:** Hong-Min Luo, Ming-Hua Du, Zhi-Long Lin, Quan Hu, Lin Zhang, Li Ma, Huan Wang, Yu Wen, Yi Lv, Hong-Yuan Lin, Yu-Li Pi, Sen Hu, Zhi-Yong Sheng

**Affiliations:** ^1^Laboratory of Shock and Organ Dysfunction, Burns Institute, the First Hospital Affiliated to the People's Liberation Army General Hospital, 51 Fu Cheng Road, Beijing 100048, China; ^2^Burns Institute, the First Hospital Affiliated to the People's Liberation Army General Hospital, 51 Fu Cheng Road, Beijing 100048, China; ^3^Obstetrics and Gynecology Department, the First Hospital Affiliated to the People's Liberation Army General Hospital, 51 Fu Cheng Road, Beijing 100048, China; ^4^Department of Critical Care Medicine, the First Hospital Affiliated to the People's Liberation Army General Hospital, 51 Fu Cheng Road, Beijing 100048, China; ^5^Department of Ophthalmology, the First Hospital Affiliated to the People's Liberation Army General Hospital, 51 Fu Cheng Road, Beijing 100048, China

## Abstract

*Objective*. Lipid peroxidation plays a critical role in burn-induced plasma leakage, and ulinastatin has been reported to reduce lipid peroxidation in various models. This study aims to examine whether ulinastatin reduces fluid requirements through inhibition of lipid peroxidation in a swine burn model. *Methods*. Forty miniature swine were subjected to 40% TBSA burns and were randomly allocated to the following four groups: immediate lactated Ringer's resuscitation (ILR), immediate LR containing ulinastatin (ILR/ULI), delayed LR resuscitation (DLR), and delayed LR containing ulinastatin (DLR/ULI). Hemodynamic variables, net fluid accumulation, and plasma thiobarbituric acid reactive substances (TBARS) concentrations were measured. Heart, liver, lung, skeletal muscle, and ileum were harvested at 48 hours after burn for evaluation of TBARS concentrations, activities of antioxidant enzymes, and tissue water content. *Results*. Ulinastatin significantly reduced pulmonary vascular permeability index (PVPI) and extravascular lung water index (ELWI), net fluid accumulation, and water content of heart, lung, and ileum in both immediate or delayed resuscitation groups. Furthermore, ulinastatin infusion significantly reduced plasma and tissue concentrations of TBARS in both immediate or delayed resuscitation groups. *Conclusions*. These results indicate that ulinastatin can reduce fluid requirements through inhibition of lipid peroxidation.

## 1. Introduction

Burn injury is one of the most severe forms of injuries that evokes both strong physical and emotional responses, producing considerable morbidity and mortality. Data supplied by the National Center for Injury Prevention and Control indicate that in 2010 there were 412,256 (133.53/100,000) nonfatal fire/burn injuries and 3,194 (1.03/100,000) fire/burn deaths in the United States [1, 2]. The cost of fire/burn injuries, including both medical costs and cost of lost productivity, is very high, costing a total of 7.546 billion dollars in 2000 [3]. Hypovolemic shock can develop rapidly after major burn injury, and current treatment for burn shock mainly focuses on maintaining a sufficient tissue perfusion with early, adequate fluid resuscitation. Currently, the most wildly used formula of fluid resuscitation in burn injury is the Parkland formula, which advocates providing 4 mL of Ringer's lactate/kg/%TBSA (total body surface area) burned/24 hours after burn, with one-half of the fluid expected to be infused over the first 8 hours and the remaining infused over the next 16 hours [4]. However, burn shock can develop and progress despite fluid resuscitation because much of the infused fluid leaks into the extravascular space, and sometimes extensive fluid resuscitation can exacerbate the interstitial edema, producing life-threatening complication such as abdominal compartment syndrome [5, 6]. Furthermore, although an early, adequate fluid resuscitation is practically achievable under normal conditions, effective treatment is a challenging issue in mass casualty incidents caused by forces of nature or by accidental or intentional explosions and conflagrations, where the environmental conditions, the presence of mass casualties, and logistic constraints preclude the availability of intensive fluid resuscitation. Thus, the development of pharmacologic resuscitation strategies that could reduce the fluid requirements for burn injury would be beneficial both in civilian burns or in burn disasters.

Previous studies have indicated that the burn-induced hypovolemic shock is mainly due to the increase in total body capillary permeability and the subsequent plasma leakage [4, 7]. Previous studies also suggest that reactive oxygen species (ROS) contribute to the increased microvascular permeability, edema formation, and tissue damage after burn injury [8–12]. After major injury, the peripheral perfusion and oxygen are decreased; however, the restoration of oxygen delivery during aggressive fluid resuscitation will initiate a deleterious cascade of events that results in the burst of oxygen radicals and causes lipid peroxidation which influences numerous cellular functions [13]. Physiological functions of cell membranes change because lipid peroxidation modifies properties of the membrane such as membrane potential, fluidity, and permeability to different substance [14]. Increased plasma and tissue levels of lipid peroxidation products, such as malondialdehyde (MDA), have been well documented after thermal injury [13, 15–20]. In addition, the use of antioxidants has been found to be efficacious in reducing fluid requirements after burn injury [21–27].

Ulinastatin is a protease inhibitor obtained from human urine, and it has been reported to have free radical-scavenging properties in various models [28–30]. Our recent study also showed that ulinastatin attenuated vasopermeability both in vivo and in vitro [31]. The present study tested the hypothesis that ulinastatin would attenuate lipid peroxidation and tissue edema, thereby reducing fluid requirement after major burn injury in a swine model.

## 2. Materials and Methods

### 2.1. Swine Burn Model and Treatment

Forty female, inbred Chinese Wuzhishan miniature swine (4–6 months, 20–25 kg, purchased from the Institute of Animal Sciences, Chinese Academy of Agricultural Sciences, Beijing, China) were used. They were acclimatized in the animal quarter of our laboratory for two weeks. All the animals were fasted for 16 h, and water was withheld for 4 h before the surgery. Under anesthesia with intramuscular injection of ketamine (Gu-Tian Pharmacy, Fu Jian Province, China), animals were then instrumented with a thermodilution catheter of PICCO-PLUS monitor (Pulsion Co., Germany) in the aorta for the measurement of hemodynamic variables, and a vascular catheter was positioned in the superior vena cava for drawing blood samples and fluid infusion. After surgery, all animals were monitored for 1 hour to assure hemodynamic stabilization, and the baseline data were then recorded. The animals were then infused with 5 mg/kg of propofol to assure adequate anesthesia and were subjected to a 40% TBSA full-thickness flam burn injury. A urinary catheter was inserted in the bladder and connected to a commercial urine collection bag. Animals were given buprenorphine (10 micrograms/kg i.v., Sigma, St. Louis, MO) immediately after burn injury and every 12 hours thereafter for sedation and pain control. The animals were kept in special slings for monitoring. All animal experiments were approved by the Committee of Scientific Research of First Hospital Affiliated to General Hospital of PLA, China, and were conducted in accordance with the National Institute of Health Guide for the Care and Use of Laboratory Animals.

The injured animals were randomly allocated to the following four groups: immediate resuscitation with lactated Ringer's (ILR), immediate resuscitation with lactated Ringer's containing ulinastatin (ILR-ULI), delayed resuscitation with lactated Ringer's (DLR); delayed resuscitation with lactated Ringer's containing ulinastatin (DLR-ULI), and with 10 pigs in each group. For ILR and ILR-ULI groups, each pig was infused with 4 mL/kg lactated Ringer's alone or lactated Ringer's containing ulinastatin (80,000 U/kg) over 30 minutes immediately after burn injury, followed by continuous infusion of lactated Ringer's, with the infusion rate adjusted each hour to maintain a urine output of 1-2 mg/kg/h. For DLR and DLR-ULI groups, each swine was initially infused with 4 mL/kg or lactated Ringer's containing ulinastatin (80,000 U/kg) over 30 minutes at 6 hours after burn, followed by continuous infusion of lactated Ringer's, with the infusion rate adjusted each hour to maintain a urine output of 1-2 mg/kg/h. 24 hours after burn injury, a second dose of 40,000 U/kg ulinastatin was given simultaneously with continuous lactated Ringer's in ILR-ULI and DLR-ULI groups. 

### 2.2. Experimental Measurements

Hemodynamics variables including mean arterial pressure (MAP), cardiac output (CO), pulmonary vascular permeability index (PVPI), and extravascular lung water index (ELWI) were measured using PICCO-PLUS monitor (Pulsion, Germany). 

Net fluid accumulation was defined as cumulative infused fluid volume minus cumulative urine output and was measured hourly as previously described by Dubick et al. [24].

Blood samples were collected for determination of hematocrit and thiobarbituric acid reactive substances (TBARS) concentration. Hematocri was determined by the clinical laboratory in our hospital. TBARS were determined as an index of lipid peroxidation using a commercial kit (Nanjing Jiancheng Science and Technology Co., Ltd, Nanjing, China) according to manufacturer's instruction. At 48 hours after burn, pigs were euthanized, and heart, liver, lung, muscle and ileum were harvested for determining concentrations of TBARS, activities of antioxidant enzymes (catalase, copper-zinc, and manganese superoxide dismutase) using commercial kits (all purchased from Nanjing Jiancheng Science and Technology Co., Ltd, Nanjing, China). Water content rate of the harvested tissues was measured in percentage of dry/wet ratio as previously described [31]. 

### 2.3. Statistical Analysis

SPSS 13.0 statistical software was used, and all results were expressed as mean ± SD. One-way ANOVA was used for comparison among all groups, followed by the Student-Newman-Keuls (SNK) test for comparison between two groups. Differences were considered to be statistically significant when *P* < 0.05.

## 3. Results

### 3.1. Hemodynamic Variables

After thermal injury, no significant changes in MAP or CO in immediate resuscitation groups were observed (Table 1). MAP and CO were decreased in the delayed resuscitation groups before resuscitation is initiated. After resuscitation, the MAP and CO in both delayed resuscitation groups were gradually restored to near baseline levels; however, the CO was slightly higher in DLR-ULI group when compared to that of DLR group (Table 1). The PVPI and ELWI were increased after thermal injury despite resuscitation; however, ulinastatin significantly attenuated the increase in PVPI and ELWI both in immediate or delayed resuscitation groups (Table 1). 

**Figure 1:**
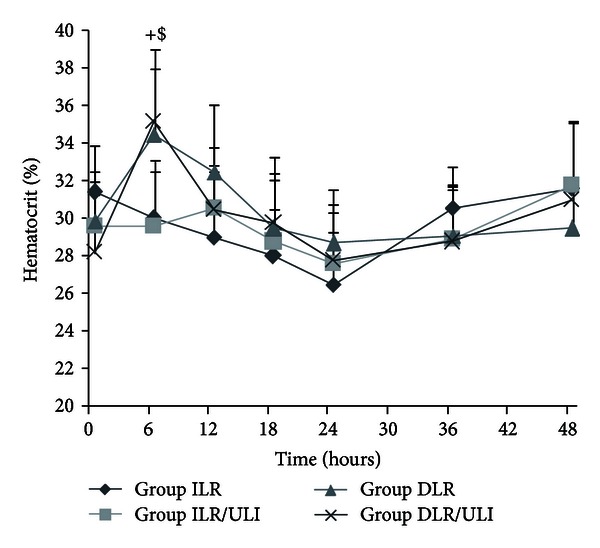
Hematocrit after thermal injury and fluid resuscitation in pigs. Data were expressed as mean ± SD. *n* = 7–10 per time point. ^+^ILR versus DLR;  ^$^ILR/ULI  versus DLR/ULI at *P* < 0.05.

There were no significant changes in hematocrit in immediate resuscitation groups after thermal injury (Figure 1). In delayed resuscitation groups, hematocrit increased after burn injury due to loss of plasma volume, however, it was returned to near baseline levels after resuscitation, showing adequate restoration of plasma volume in both groups (Figure 1).

### 3.2. Effect of Ulinastatin on Net Fluid Accumulation

**Figure 2:**
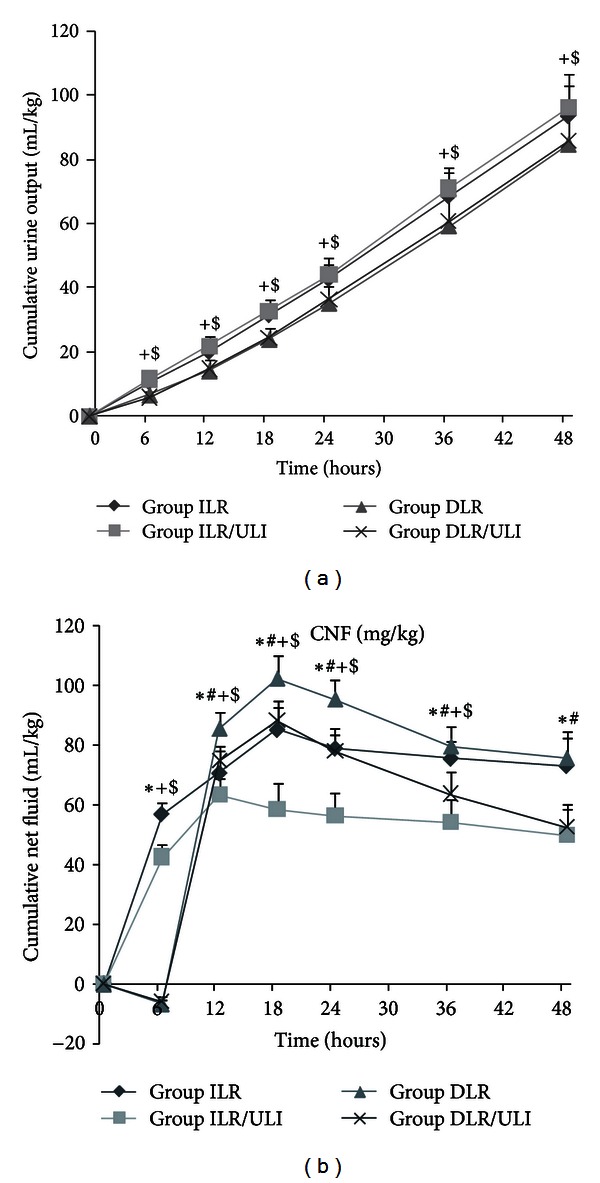
Accumulative urine output (a) and net fluid accumulation (b) after thermal injury and fluid resuscitation in pigs. Data were expressed as mean ± SD. *n* = 7–10 per time point. *ULI versus ILR; ^#^DLR/ULI versus DLR; ^+^ILR versus DLR; and ^$^ILR/ULI versus DLR/ULI at *P* < 0.05.

After thermal injury, pigs were given either immediate or delayed resuscitation with adjusted infusion rate to maintain urine output between 1 and 2 mL/kg/h. Burn injury resulted in a decrease in urine outputs in both delayed resuscitation groups before resuscitation; however, when resuscitation initiated, urine outputs were similar in all groups (Figure 2(a)). Resuscitation resulted in different extent of net fluid accumulation in all groups; however, ulinastatin significantly reduced net fluid accumulation both in immediate and delayed resuscitation groups (Figure 2(b)). 

### 3.3. Effect of Ulinastatin on TBARS Concentrations and Antioxidant Enzymes Activities

**Figure 3:**
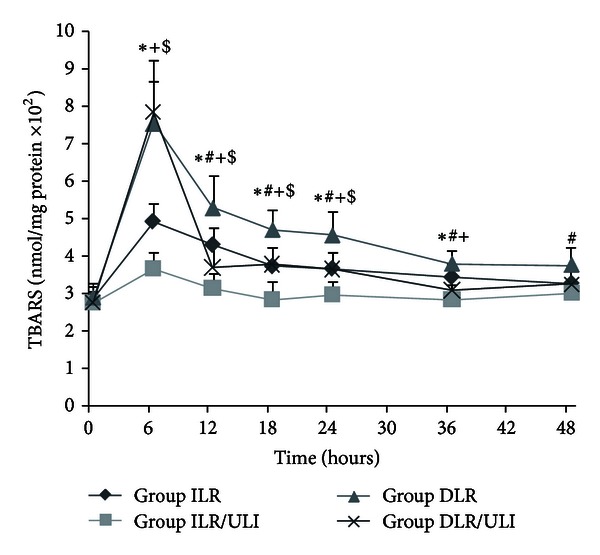
Plasma TBARS concentrations after thermal injury and fluid resuscitation in pigs. Data were expressed as mean ± SD. *n* = 7–10 per time point. *ULI versus ILR; ^#^DLR/ULI versus DLR; ^+^ILR versus DLR; and ^$^ILR/ULI versus DLR/ULI at *P* < 0.05.

**Figure 4:**
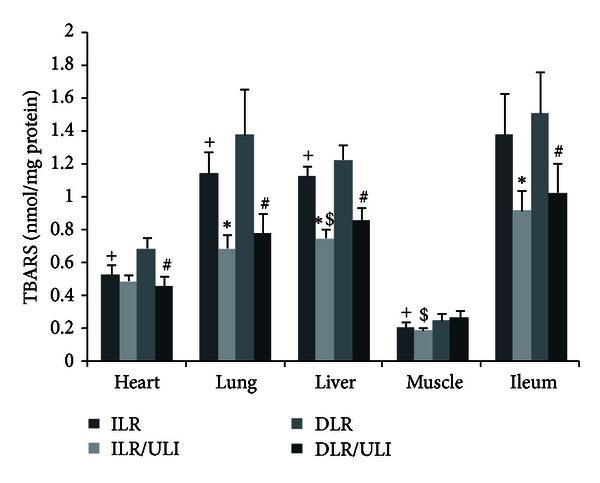
TBARS concentrations in heart, liver, lung, muscle, and ileum 48 hours after thermal injury in pigs. Data were expressed as mean ± SD. *n* = 7–10 in each group. *ULI versus ILR; ^#^DLR/ULI versus DLR; ^+^ILR versus DLR; and ^$^ILR/ULI versus DLR/ULI at *P* < 0.05.

Burn insult and resuscitation resulted in an increase in plasma TBARS concentrations, which was more prominent in delayed resuscitated animals early after burn (Figure 3). Ulinastatin significantly prevented the increase in plasma TBARS both in immediate and delayed resuscitation groups (Figure 3). The TBARS concentrations in different organs, especially in lung and liver tissues, were lower in ulinastatin-treated animals, although some of them did not reach statistical significance (Figure 4). However, activities of antioxidant enzymes (superoxide dismutase and catalase) in heart, liver, lung, skeletal muscle, and ileum were similar in all groups (Table 2).

### 3.4. Effect of Ulinastatin on Water Content in Different Organs

**Figure 5:**
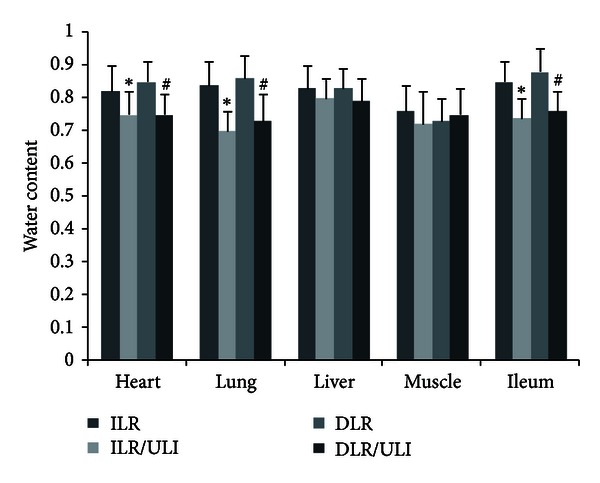
Water content in heart, liver, lung, muscle and ileum 48 hours after thermal injury in pigs. Data were expressed as mean ± SD. *n* = 7–10 in each group. *ULI versus ILR; ^#^DLR/ULI versus DLR at *P* < 0.05.

Ulinastatin significantly reduced the water content of heart, lung, and ileum in both immediate or delayed resuscitation groups (Figure 5). However, there was no significant difference among all groups in the water content of liver and skeletal muscle tissues (Figure 5).

## 4. Discussion

Hypovolemic shock is a key factor influencing the mortality rate during the early phase of major burn injury. Current efforts to improve burn shock outcome mainly focus on early and adequate fluid resuscitation. Intensive fluid resuscitation, however, may exacerbate interstitial edema and cause serious adverse events, such as abdominal compartment syndrome. Furthermore, intensive fluid resuscitation from burn shock is usually difficult in austere environments (battlefield, forest conflagration, or earthquake) due to the environmental conditions, the presence of mass casualties, and logistic constraints. Thus, pharmacologic agents that could reduce the fluid requirements for burn injury by attenuating plasma leakage would benefit casualties both in civilian burns or in burn disasters.

Emerging evidence suggests that lipid peroxidation after thermal injury plays a critical role in the increase in vascular permeability and the subsequent plasma leakage [4]. Ulinastatin is a protease inhibitor obtained from human urine, and it has been reported to reduce lipid peroxidation in various models [29, 32–35]. Our recent study also showed that ulinastatin attenuated vasopermeability both in vivo and in vitro [31]. However, it remains unknown whether ulinastatin treatment would reduce lipid peroxidation and fluid requirements in swine model of major burn injury.

In this study we adopted a swine model of 40% TBSA burn injury to investigate the effects of ulinastatin on lipid peroxidation and fluid requirements. We have shown that in this swine burn model, ulinastatin treatment attenuates lipid peroxidation, tissue edema, and net fluid accumulation and thereby reducing fluid requirements.

Ulinastatin is a relatively safe drug, and the dosages from 5,000 to 1,000,000 U/kg have been reported in different animal models [36–38]. In this study, we used a high dosage of ulinastatin (80,000 U/kg in the first 24 hours after burn and then another 40, 000 U/kg in the second 24 hours after burn) in order to obtain more significant protective effects.

We first evaluated the effects of ulinastatin on fluid requirements in burnt pigs followed by immediate resuscitation or delayed resuscitation in an adjusted rate according to urine output. The hemodynamic response to burn injury and resuscitation was similar to previous reports [24, 39, 40]. There was no significant changes in MAP and CO in immediate resuscitation groups. Although there was a reduction in MAP and CO in delayed resuscitation groups after burn injury, they were gradually restored to near baseline levels when resuscitation initiated. Similarly, hematocrit increased after burn injury due to loss of plasma volume, however, it was returned to near baseline levels after resuscitation in delayed resuscitation groups. These results suggest that adequate restoration of plasma volume was achieved in both resuscitation strategies. An increase in PVPI and ELWI was observed after thermal injury despite resuscitation, however, ulinastatin significantly attenuated the burn-induced increase in PVPI and ELWI both in immediate or delayed resuscitation groups. This suggests that ulinastatin could attenuate burn-induced lung injury and edema which was supported by other studies [41, 42]. PVPI and ELWI in DLR and DLR/ULI groups were significantly higher than those in ILR and ILR/ULI groups at 6 hours after injury when DLR and DLR/ULI groups were not given fluid resuscitation. It is possible that ELWI and PVPI are overestimated by PiCCO system because of hypovolemia which is one of the limitations of PiCCO system [43].

In this experiment, the urine output was maintained at 1-2 mL/kg/h by adjusting the infusion rate. Urine output was similar in animals treated with or without ulinastatin. However, ulinastatin significantly attenuated the net fluid accumulation in both immediate and delayed resuscitation groups. Furthermore, water content of heart, lung, and ileum was significantly reduced in ulinastatin-treated animals. These findings, together with previous studies by ours and others [31, 42], indicate that ulinastatin is able to attenuate the burn-induced increase in vascular permeability and plasma volume loss and thereby reducing fluid requirements.

Since free radical-induced lipid peroxidation is suggested to be implicated in burn-induced increase in vascular permeability and the subsequent plasma leakage [4], ulinastatin has been reported to reduce lipid peroxidation in various models, including burn models [29, 32–35, 44]. Thus, we further investigated whether the protective effects of ulinastatin on burn-induced increase in vascular permeability and plasma volume loss were associated with reduced lipid peroxidation. We measured the plasma and tissue concentrations of TBARS as an index of lipid peroxidation. In consistent with previous study [44], we found that burn insult that resulted in an increase in lipid peroxidation and ulinastatin administration effectively attenuated the burn-induced lipid peroxidation. We further measured the antioxidant enzymes activities in heart, liver, lung, muscle, and ileum harvested at 48 hours after burn. However, in contrast to previous investigation by Shimazaki et al. [44], no significant difference in antioxidant enzymes activities was observed among all groups. Differences between Shimazaki's results and ours could be due to differences in animals, tissues, or time of tissue harvest, and further study is needed to confirm the effects of ulinastatin on antioxidant enzymes activities.

## 5. Conclusions

In summary, ulinastatin, a protease inhibitor, attenuates burn-induced increase in vascular permeability and net fluid accumulation and has a therapeutic role in reducing fluid requirements of thermal injuries. These protective effects of ulinastatin may be mediated in part through the inhibition of burn-induced lipid peroxidation. This study offers a potential small-volume fluid resuscitation strategy in combating major burn injury. 

## Figures and Tables

**Table 1:** Hemodynamic variables in MAP, CO, ELWT, and PVPI.

Variables	After injury (h)						
	0	6	12	18	24	36	48
MAP (mm Hg)							
Group ILR	90.5 ± 6.5	90.2 ± 5.8^c^	93.6 ± 6.3	91.2 ± 6.1	92.3 ± 6.5	92.6 ± 6.4	96.2 ± 4.9
Group ILR/ULI	89.3 ± 4.4	93.2 ± 6.4^d^	94.6 ± 5.5	94.2 ± 6.3	93.3 ± 5.2	90.1 ± 4.3	94.0 ± 3.3
Group DLR	91.1 ± 6.6	75.2 ± 3.4	89.2 ± 5.3	96.0 ± 6.2	92.0 ± 5.3	96.3 ± 6.9	93.8 ± 6.8
Group DLR/ULI	90.7 ± 5.3	73.2 ± 4.6	90.6 ± 4.5	91.0 ± 5.7	95.0 ± 5.9	95.1 ± 6.8	94.5 ± 5.5
CO (L·min^−1^)							
Group ILR	3.76 ± 0.43	3.66 ± 0.38^c^	3.39 ± 0.46^c^	3.61 ± 0.34^c^	3.88 ± 0.49^c^	3.78 ± 0.45	3.79 ± 0.47
Group ILR/ULI	3.69 ± 0.44	3.70 ± 0.39^d^	3.55 ± 0.36^d^	3.58 ± 0.35^d^	4.06 ± 0.69	3.87 ± 0.52	3.83 ± 0.57
Group DLR	3.89 ± 0.53	2.66 ± 0.35	2.87 ± 0.40	2.86 ± 0.35	3.07 ± 0.36	3.35 ± 0.45	3.69 ± 0.50
Group DLR/ULI	3.76 ± 0.45	2.70 ± 0.38	3.05 ± 0.28	3.18 ± 0.33^b^	3.66 ± 0.28^b^	3.67 ± 0.41	3.80 ± 0.49
ELWI (mL·kg^−1^)							
Group ILR	8.13 ± 0.64	11.17 ± 1.15^c^	11.65 ± 1.54	10.05 ± 1.09^c^	9.98 ± 0.87	9.56 ± 1.05	9.23 ± 0.56
Group ILR/ULI	8.02 ± 0.62	11.38 ± 1.24^d^	10.22 ± 0.98^a^	9.46 ± 1.07	9.23 ± 0.85^a^	8.76 ± 0.95^a^	8.48 ± 0.63^a^
Group DLR	8.15 ± 0.81	13.02 ± 0.86	12.55 ± 1.05	11.89 ± 1.12	10.26 ± 0.57	9.84 ± 0.67	9.68 ± 0.76
Group DLR/ULI	8.14 ± 0.69	13.26 ± 1.04	11.18 ± 0.78^b^	10.06 ± 0.75^b^	9.12 ± 0.60^b^	8.86 ± 0.54^b^	8.55 ± 0.57^b^
PVPI							
Group ILR	2.71 ± 0.26	3.31 ± 0.37^c^	3.78 ± 0.30	3.87 ± 0.37	3.82 ± 0.29	3.68 ± 0.35	3.25 ± 0.31
Group ILR/ULI	2.58 ± 0.25	3.28 ± 0.25^d^	3.32 ± 0.29^a^	3.13 ± 0.34^a^	3.01 ± 0.33^a ^	2.98 ± 0.21^a^	2.90 ± 0.29^a^
Group DLR	2.57 ± 0.24	4.29 ± 0.51	3.99 ± 0.27	3.78 ± 0.32	3.67 ± 0.34	3.64 ± 0.18	3.44 ± 0.31
Group DLR/ULI	2.68 ± 0.18	4.11 ± 0.14	3.50 ± 0.26^b^	3.24 ± 0.11^b^	3.17 ± 0.29^b^	3.02 ± 0.35^b^	2.75 ± 0.23^b^

MAP: mean arterial pressure, CO: cardiac output, ELWI: extravascular lung water index, PVPI: pulmonary vascular permeability index. Data were expressed as mean ± SD. *n* = 7–10 per time point. ^a^ULI versus ILR; ^b^DLR/ULI versus DLR; ^c^ILR versus DLR; and ^d^ILR/ULI versus DLR/ULI at *P* < 0.05.

**Table 2:** Effect of ulinastatin on tissue antioxidant enzyme activities.

Variables	Group	Organs				
		Heart	Lung	Liver	Muscle	Ileum
Cu-Zn SOD^#^	ILR	3.4 ± 0.5	1.3 ± 0.2	16.3 ± 2.2	1.0 ± 0.2	3.2 ± 0.5
	ILR/ULI	3.5 ± 0.6	1.2 ± 0.2	17.3 ± 2.5	0.9 ± 0.1	3.1 ± 0.4
	DLR	3.8 ± 0.2	1.2 ± 0.3	17.6 ± 1.4	1.1 ± 0.2	2.9 ± 0.4
	DLR/ULI	3.2 ± 0.6	1.0 ± 0.3	16.8 ± 1.9	1.0 ± 0.1	3.0 ± 0.3

MnSOD^+^	ILR	7.9 ± 1.3	1.7 ± 0.3	6.6 ± 0.8	1.2 ± 0.1	2.2 ± 0.3
	ILR/ULI	8.6 ± 1.5	1.5 ± 0.3	6.8 ± 0.8	1.3 ± 0.3	2.1 ± 0.4
	DLR	8.4 ± 1.4	1.5 ± 0.2	6.3 ± 0.9	1.4 ± 0.2	2.2 ± 0.3
	DLR/ULI	8.3 ± 1.4	1.6 ± 0.4	6.6 ± 0.7	1.3 ± 0.2	2.4 ± 0.3

Catalase	ILR	42.6 ± 5.6	54.3 ± 6.2	485.2 ± 73.1	8.6 ± 1.1	34.0 ± 6.5
	ILR/ULI	46.6 ± 8.3	57.0 ± 7.5	428.8 ± 63.7	7.6 ± 1.4	37.4 ± 8.5
	DLR	47.3 ± 6.1	56.3 ± 4.6	523.5 ± 59.5	8.6 ± 1.3	32.5 ± 4.4
	DLR/ULI	42.6 ± 5.7	54.3 ± 6.2	489.3 ± 76.5	8.6 ± 1.4	32.6 ± 5.3

Data were expressed as mean ± SD of units per milligram of protein. *n* = 7–10 in each group.

^#^Copper zinc superoxide dismutase.

^+^Manganese superoxide dismutase.
